# Review of poliovirus modeling performed from 2000 to 2019 to support global polio eradication

**DOI:** 10.1080/14760584.2020.1791093

**Published:** 2020-08-01

**Authors:** Kimberly M. Thompson, Dominika A. Kalkowska

**Affiliations:** Kid Risk, Inc, Orlando, FL, USA

**Keywords:** Poliovirus, eradication, modeling

## Abstract

**Introduction:**

Over the last 20 years (2000-2019) the partners of the Global Polio Eradication Initiative (GPEI) invested in the development and application of mathematical models of poliovirus transmission as well as economics, policy, and risk analyses of polio endgame risk management options, including policies related to poliovirus vaccine use during the polio endgame.

**Areas covered:**

This review provides a historical record of the polio studies published by the three modeling groups that primarily performed the bulk of this work. This review also systematically evaluates the polio transmission and health economic modeling papers published in English in peer-reviewed journals from 2000 to 2019, highlights differences in approaches and methods, shows the geographic coverage of the transmission modeling performed, identified common themes, and discusses instances of similar or conflicting insights or recommendations.

**Expert opinion:**

Polio modeling performed during the last 20 years substantially impacted polio vaccine choices, immunization policies, and the polio eradication pathway. As the polio endgame continues, national preferences for polio vaccine formulations and immunization strategies will likely continue to change. Future modeling will likely provide important insights about their cost-effectiveness and their relative benefits with respect to controlling polio and potentially achieving and maintaining eradication.

## 1. Introduction

The partners of the Global Polio Eradication Initiative (GPEI) began investing resources (both financial and human) in the early 2000s in the development and application of mathematical models of poliovirus transmission as well as economics, policy, decision, and risk analyses of polio endgame risk management options to support decisions, including vaccine policy choices. Over the last 20 years (2000–2019), three modeling groups who access GPEI data as part of a data-sharing agreement created in 2013 (i.e. Kid Risk, Inc. (KRI, Orlando, FL), Imperial College (IC, London, United Kingdom), and the Institute for Disease Modeling (IDM, Seattle, WA)) performed the bulk of this polio modeling. The three groups largely work independently, which provides some confidence to the GPEI partners when the results from the groups agree. However, sometimes the groups provide conflicting results and recommendations. In addition, some other polio modeling papers also appeared in the published literature during this time.

Modeling poliovirus transmission can quickly become complex due to the three stable serotypes (i.e. 1, 2, and 3) and numerous strains. Live polioviruses (LPVs) exist in many forms, including wild polioviruses (WPVs), live, attenuated oral poliovirus vaccine (OPV) strains, and OPV-related strains associated with evolution of the virus as OPV transmits through populations, causes secondary infections, and loses its attenuating mutations. OPV transmission can lead to the development of circulating vaccine-derived polioviruses (cVDPVs), which result from the spread of OPV-related viruses in populations with low immunization coverage until the transmitting strains become fully reverted and behave like homotypic WPVs. In addition, in some rare instances, individuals with some B-cell-related primary immunodeficiencies can develop prolonged or chronic OPV infections, which evolves over the course of their infections, and they can potentially excrete (i.e. immunodeficiency-associated VDPVs (iVDPVs)). Consistent with no evidence of poliovirus transmission through a nonhuman vector and no environmental reservoir, transmission modeling focuses on person-to-person spread, with some models distinguishing between fecal-oral and oropharyngeal routes. All LPVs pose some risk of causing paralysis in fully susceptible individuals, although the probabilities (i.e. paralysis to infection ratios (PIRs)) range from substantial (i.e. on the order of 1 chance per 200 for WPVs) to very small (i.e. on the order of 1 chance per 1,000,000 for OPV). Paralysis cases that occur in fully susceptible OPV vaccine recipients or close contacts are called vaccine-associated paralytic polio (VAPP) cases. Notably, the PIRs (e.g. VAPP rates) and the transmissibility of LPV strains, as measured by their basic reproduction numbers (R_0_s), differ by serotype and strain. In addition, an inactivated poliovirus vaccine (IPV) offers a second vaccine option, which can be given instead of or in addition to OPV. Both OPV and IPV appear to offer lifelong protection from paralysis after a single successful dose, although not every dose ‘takes’ and for OPV some competition can exist between the serotypes in multivalent formulations. Unlike for OPV, IPV recipients do not become infected with the vaccine strain. Consequently, they do not develop mucosal immunity and they cannot spread the vaccine secondarily (i.e. receipt of the IPV dose only protects the recipient). Adding even more complexity, individual immunity can wane and individuals can become reinfected and participate in transmission, with differences in the probabilities of infection and duration of excretion depending on the nature of their prior immunity. Although for most of the history of its use OPV included all three serotypes (i.e. trivalent OPV or tOPV), licensed formulations of monovalent OPV (i.e. mOPV) exist for each serotype (i.e. mOPV1, mOPV2, and mOPV3), and licensed bivalent OPV (i.e. bOPV) contains OPV for serotypes 1 and 3. The global certification of serotype 2 WPV (i.e. WPV2) eradication led to the globally coordinated cessation of serotype 2-containing OPV (i.e. OPV2) in 2016, which led countries that used tOPV prior to that time to switch to bOPV. As an inactivated vaccine, all IPV includes all three serotypes. Finally, individuals can receive vaccine either through routine immunization (RI), which follows a national schedule that delivers doses to children as they reach target ages and/or supplementary immunization activities (SIAs), which deliver doses to all individuals within a target age range over a short period of time, typically independent of prior immunization. SIAs include large, planned, and preventive SIAs (pSIAs) or reactive, outbreak response SIAs (oSIAs). As of early 2020, only serotype 1 WPV (i.e. WPV1) continues indigenous transmission (and only in Pakistan and Afghanistan), and global certification of serotype 3 WPV (WPV3) eradication occurred in October 2019. Since OPV2 cessation in 2016, serotype 2 cVPDVs (i.e. cVDPV2 s) have arisen in multiple countries despite pre-OPV2 cessation efforts to prevent them. Responses to these cVDPV2 outbreaks using mOPV2 imply ongoing transmission of OPV2-related strains.

Given the complexity of poliovirus immunity and multiple vaccine options, transmission and health economic modeling can provide insights that can support decision-makers as they evaluate different decisions and policy options. The GPEI partners implicitly value this type of modeling by engaging multiple modeling groups, and some prior studies documented the important role of modeling with respect to supporting some GPEI decisions [1–4]. Notably, however, we could not identify a comprehensive list of GPEI decisions, which makes it difficult to systematically document the decision-support provided by modeling. In addition, no systematic or comprehensive review of the polio modeling literature performed to support polio endgame risk management currently exists, and the different modeling groups tend to cite their own work (including the authors of this review) with limited reference to the independent work of the other groups. Furthermore, the published literature also includes relevant modeling studies by other authors. We sought to document the polio studies published by the different groups and to systematically review the nature of the polio transmission and economic modeling papers published in English for 2000–2019. Section 2 describes the methods we used to identify, code, and evaluate the literature. Section 3 summarizes the results of the systematic review. Section 4 provides a historical record of all of the polio-related studies published by the three GPEI-supported groups and summarizes the polio transmission models and economic models that we identified by other authors. Section 5 discusses cross-cutting themes addressed by multiple studies. Section 6 highlights differences in modeling approaches and methods. Sections 7 and 8 provide conclusions and expert opinion.

## 2. Methods

**Figure 1.**
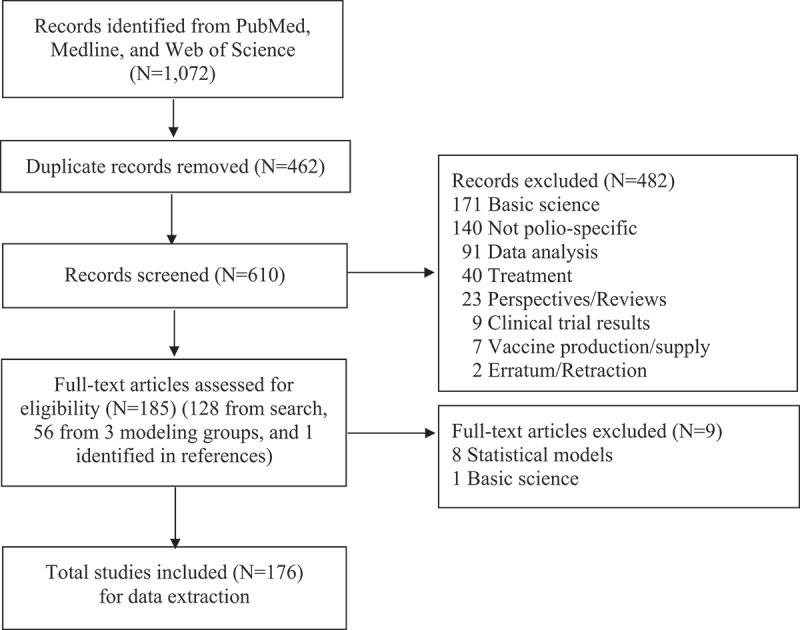
Literature search process.

We searched Web of Science (Clarivate Analytics, Philadelphia, PA) and PubMed/Medline (United States National Library of Medicine, Bethesda, MD) for papers published in English after 1 January 2000 and before 31 December 2019 that included a combination of the terms: ‘polio*’ and ‘model*’ in their titles or abstracts. We screened the titles and abstracts of the search results to create a database of all studies that included mathematical modeling of poliovirus transmission and/or the economic evaluation of policies for risk management in the polio endgame. We evaluated the full text of papers for which the abstract did not contain sufficient information, and we excluded papers that did not include modeling of poliovirus transmission and/or economic analyses. We also reviewed the websites of the three modeling groups that support the GPEI partners: KRI [5], IC [6], and IDM [7]. We then added any missing polio-specific publications by the three GPEI-partner-supported modeling groups, including any that did not report on mathematical models of poliovirus transmission and/or economics related to risk management for the polio endgame. We included these papers to summarize the complete body of polio-related work published by the three modeling groups for 2000–2019. Figure 1 summarizes the search process.

We focused this review on the papers that reported the results of mathematical modeling of poliovirus transmission or economic analysis or their combination (i.e. integrated models). For each paper, we extracted the modeling group (i.e. KRI, IC, IDM, or other), publication year, and characterized the type of modeling performed or information reported. We applied the following hierarchy for characterization: (i) integrated modeling (i.e. including both dynamic transmission and economic modeling), (2) dynamic transmission models, subcategorized as a differential-equation-based (DEB), stochastic compartmental (SC), individual-based (IB), and/or discrete-event simulation (DES), (3) economic analyses, or (4) other, which only applied to some publications by the three GPEI-supported modeling groups, and which we only included to provide a historical record of the publications of these groups. We categorized the group of other papers as statistical or meta-analyses, which we subcategorized according to their focus on estimating risks, poliovirus transmission characteristics, vaccine effectiveness, or mucosal immunity, or as discussions of policy options, reviews, perspectives, or commentaries. This review excluded statistical analyses (e.g. exploration of risk factors, time-series analyses of incidence data) and discussions of policies, reviews perspectives, or commentaries, except those published by one of the three GPEI-supported modeling groups. As part of our review, we also identified topics of interest to the GPEI partners, which we found discussed by more than one of the three modeling groups.

## 3. Results

Following the search process shown in Figure 1, the systematic literature review and addition of other studies by the three GPEI-supported modeling groups led to the extraction of information from 176 included studies [1-4, 8-179]. As noted, during review of the full text of the studies identified by the search, we excluded papers that presented statistical analyses that did not include a mechanistic poliovirus transmission model [180–187].

Table 1 summarizes some attributes of the included studies. Not surprisingly, the number of publications by each modeling group reflects the beginning of their efforts (i.e. KRI 78 papers since 2003, IC 46 papers since 2006, and IDM 19 papers since 2014). Similarly, as the number of modeling groups increased, so did the number of publications per 5-year time period (i.e. 5 papers 2000–2004, 22 papers 2005–2009, 45 papers 2010–2014, and 103 papers from 2015 to 2019). All of the modeling groups developed and applied some dynamic transmission models, although the extent of these efforts varied considerably. For example, only KRI combined dynamic transmission and economic modeling into integrated policy models and used all of the different types of dynamic transmission modeling tools (i.e. DEB, SC, IB, and DES). In addition, the three modeling groups tend to preferentially apply different modeling tools (i.e. DEB modeling dominates for KRI, SC for IC, and IB for IDM). We did not include studies that performed statistical simulation of infections (e.g. [136]) as dynamic transmission models. As shown in Table 1, all of the modeling groups also published papers that did not include transmission modeling or economic analyses. Notably, IC invested considerable efforts in characterizing vaccine effectiveness based on clinical trial and surveillance data, and on characterizing risks using statistical epidemiology to support inferences. Table 1 shows multiple reviews performed by all of the modeling groups to develop inputs for their transmission models. Table 1 also includes the contributions to the literature from others, which largely represent single papers, but with notable exception of multiple papers by Professor James Koopman (University of Michigan).

**Table 1.** Characteristics of included peer-reviewed polio-related studies published in English 2000–2019.

Characteristic	
Modeling group	KRI (n = 78) [1-4, 8-81] IC (n = 46) [82–127] ^a,b^ IDM (n = 19) [128–146] Poliovirus transmission modeling by others (n = 24) [147–171] Economic analyses by others (n = 9) [172–179]
Publication date	2000–2004 (n = 5) [8, 147, 148, 172, 173] 2005–2009 (n = 22) [9-22, 82–86, 149, 150, 174, 175] 2010–2014 (n = 45) [1, 2, 23–42, 87–98, 128–131, 151–154, 176, 177] 2015–2020 (n = 103) [3, 4, 43–81, 99–146, 155–171, 178, 179]
Publication type	Integrated (DEB transmission and economic combined) (n = 12) [9, 18–20, 25, 51, 54, 59, 61, 62, 64, 65] Dynamic transmission only (n = 70) ^c,d^ DEB (n = 45) [10, 14, 22, 26, 33–36, 38–40, 43, 47, 49, 52, 53, 55–58, 60, 68, 69, 73, 74, 77, 97, 147–157, 160, 162–166, 171] ^c^ SC (n = 15) [27, 46, 70, 71, 75, 76, 97–100, 147, 158, 161, 167–169] IB (n = 10) [24, 41, 129, 132–135, 155, 159, 170]^d^ DES,DEB (n = 3) [4, 50, 81] Economic/cost analysis only (n = 15) [11, 12, 21, 23, 66, 78, 146, 172–179] Statistical analyses (by 3 GPEI-supported modeling groups only) (n = 38) Risk assessment (n = 19) [44, 93, 101, 102, 109–113, 116, 117, 130, 131, 136, 140–143, 145] Vaccine effectiveness (n = 17) [82–84, 87–92, 103–108, 114, 115] Mucosal immunity (n = 2) [85, 86] Reviews (by 3 GPEI-supported modeling groups only) (n = 14) Transmission model inputs (n = 11) [13, 29, 30, 32, 94, 96, 126–128, 138, 139] Risk model inputs (n = 3) [67, 72, 125] Discussions (by 3 GPEI-supported modeling groups only) (n = 26) Policy options (n = 5) [8, 28, 31, 37, 80] Perspectives (n = 13) [1-3, 15–17, 45, 63, 95, 122, 124, 137, 144] Commentaries (n = 8) [42, 48, 79, 118–121, 123]

Abbreviations: DEB, differential-equation-based model; DES, discrete-event simulation model; IB, individual-based model; IC, Imperial College; IDM, Institute for Disease Modeling; IPV, inactivated poliovirus vaccine; iVDPVs, immunodeficiency-associated vaccine-derived poliovirus; KRI, Kid Risk, Inc.; OPV, oral poliovirus vaccine; SC, stochastic compartmental model; SIAs, supplementary immunization activities.

Notes

^a^Two papers included one middle author from IDM [100, 109].

^b^One author on three papers now at the London School of Hygiene and Tropical Medicine [116, 117, 127].

^c^Two papers included both DEB and SC model formulations [97, 147].

^d^One paper included both DEB and IB model formulations [155].

Table 2 provides an overview of some of the attributes of the model structures and assumptions for the 83 papers that included a poliovirus transmission model [4, 9, 10, 14, 18–20, 22, 24–27, 33–36, 38–41, 43, 46, 47, 49–62, 64, 65, 68–71, 73–77, 81, 97–100, 129, 132–135, 147–171] organized by modeling group. Mathematical models for poliovirus transmission vary considerably in their complexity. The review identified papers that ranged from analytical exploration of theoretical issues using hypothetical populations for an average poliovirus to papers that simulated all of the complexity that comes with seasonal transmission of three serotypes of LPVs in populations with complicated national immunization strategies and histories. Table 2 shows the counts of and references for papers that modeled the transmission of outbreak viruses only, transmission of WPV, cVDPV, and/or OPV viruses, and those that included endogenous OPV evolution and model all LPVs. Table 2 also identifies the papers that included different attributes, including consideration of seasonality, specific-serotype transmission model inputs, OPV secondary spread, VAPP, both fecal-oral and oropharyngeal transmission routes, waning immunity, reinfection, and/or boosting OPV-induced immunity by IPV. With respect to mixing, Table 2 also captures whether each model included more than one age group and/or subpopulation and whether it included heterogeneous preferential mixing between age groups and/or subpopulations. With highly variable model structures, Table 2 identifies papers that included multiple immunity states to account for differences in immunity induced by OPV and IPV (in some cases as a function of the dose history), and immunity derived from maternal antibodies in infants. Table 2 also noted the papers with models that included one or more latent (i.e. infected but not infectious) stages and whether the models used a multi-stage infection process. DEB transmission models with a single stage for infection can lead to unrealistically short durations for many infections and long tails for others [188], which motivates the use of multi-stage infection processes in DEB models. SC models can avoid the issue of exponential departure rates from a single infection stage by using distributions instead of multiple stages (i.e. they simulate multi-stage infection processes more directly), and IB models may use time-varying functions for individual agents to model infections. DEB models can be solved analytically for some simple models or simulated numerically. SC models involve different types of stochastic simulation, which include following every single transition that occurs in the population with variable time steps [189], or using draws from an appropriate probability distribution (e.g. Poisson) to randomly determine the number of transitions that occur in the system during a fixed time step [188]. IB models simulate individual agents, and DES models track events. Remarkably, the review also identified a few theoretical papers that included an environmental reservoir, which is not consistent with the epidemiological experience with polioviruses. Finally, Table 2 also provides a high-level perspective on the types of immunization included in each paper by noting the studies that included OPV in RI, OPV in SIAs, IPV in RI, and IPV in SIAs, the studies that account for differences between various IPV and OPV RI schedules, and that account for the reality of repeatedly missing the same children during successive SIAs.

**Table 2.** Numbers of papers with specific characteristics of dynamic transmission models by group among 83 papers with such models.

Characteristic	KRI	IC	IDM	Other
**Transmission models**	49 a,b [10, 27, 70]	4 [97–100]	5 [129, 132–135]	24 [147–171]
WPV, cVDPV, and/or OPV outbreaks (only)	1 [70]	3 [97–99]	1 [132]	9 [147, 148, 152, 157–159, 167–170]
WPV, cVDPV, and/or OPV transmission	11 a [10, 27]	1 [100]	3 [129, 134, 135]	13 [149–151, 154, 156, 160–166, 171]
All LPVs transmission and OPV evolution	37 b		1 [133]	2 [153, 155]
**Models that include specific complexities**				
Seasonality	47 a,b [10]		1 [132]	4 [158, 160, 162, 163, 166]
Specific-serotype transmission model inputs	39 b [10, 27]	3 [98–100]	5 [129, 132–135]	5 [161, 162, 165, 166, 170]
OPV secondary spread	48 a,b [10, 27]	1 [100]	4 [129, 133–135]	8 [151, 153–155, 161, 165, 166, 170]
VAPP	46 a,b			1 [150]
Fecal-oral and oropharyngeal transmission separately	37 b			
Waning	46 a,b		3 [129, 134, 135]	3 [154, 163, 165]
Reinfection	46 a,b		3 [129, 134, 135]	3 [154, 163, 165]
Boosting of immunity by IPV	46 a,b		3 [134, 135]	
Multiple age groups	45 c	1 [98]	3 [129, 132, 134]	5 [149, 154, 158, 159, 165, 170]
Subpopulations	34 d		2 [129, 132]	4 [159, 160, 163, 164]
Heterogeneous preferential mixing between age groups	45 c	1 [98]	1 [132]	1 [159]
Heterogeneous preferential mixing between subpopulations	34 d		2 [132, 133]	2 [159, 163]
**Models that include specific states**				
Different immunity states for OPV and IPV if model includes both	48 a,b [10, 27]			3 [153, 161, 163]
Multiple immunity states for immunity induced for different OPV and/or IPV dose histories	37 b		5 [129, 132–135]	1 [162]
Maternal antibodies in infants	37 b		5 [129, 132–135]	1 [158]
1 or more latent stages (infected not infectious)	48 a,b [10, 27]	3 [97–99]		9 [151, 152, 156, 159, 161–163, 166, 170]
Multi-stage infection processes	38 b [27]		5 [129, 132–135]	2 [151, 161]
Environmental reservoir				3 [149, 160, 171]
**Vaccination considered**				
OPV in RI	48 a,b [10, 27]	1 [100]	5 [129, 132–135]	13 [150, 151, 153–157, 159, 161, 163–165, 171]
OPV in SIAs	45 b [10, 14, 18–20, 22, 24, 25]	2 [98, 100]	5 [129, 132–135]	8 [150, 151, 155, 159, 160, 162, 166, 170]
IPV in RI	38 b [10]	1 [97]	2 [133–135]	8 [150, 152, 153, 159, 161, 163, 164, 170]
IPV in SIAs	9 [51, 55, 59, 64, 68, 73–76]		1 [133]	1 [150]
Differences in OPV and IPV RI schedules	37 b		5 [129, 132–135]	
Repeatedly missed children in successive SIAs	37 b	1 [100]		

Abbreviations: cVDPV, circulating vaccine-derived poliovirus; IC, Imperial College; IDM, Institute for Disease Modeling; IPV, inactivated poliovirus vaccine; KRI, Kid Risk, Inc.; LPV, live poliovirus; OPV, oral poliovirus vaccine; RI, routine immunization; SIAs, supplementary immunization activities; VAPP, vaccine-associated paralytic polio; WPV, wild poliovirus.

a All of the following: [9, 14, 18–20, 22, 24–26].

b All of the following: [4, 33–36, 38–41, 43, 46, 47, 49–62, 64, 65, 68, 69, 71, 73–77, 81].

c All of the following: [4, 9, 10, 14, 18–20, 22, 24, 26, 33–36, 38–41, 43, 46, 47, 49–62, 64, 65, 68, 71, 73–77, 81].

d All of the following: [4, 10, 26, 35, 36, 40, 43, 46, 47, 49–62, 64, 65, 68, 69, 71, 73–77, 81].

Table 3 summarizes the populations considered by the 83 papers that included a polio transmission model [4, 9, 10, 14, 18–20, 22, 24–27, 33–36, 38–41, 43, 46, 47, 49–62, 64, 65, 68–71, 73–77, 81, 97–100, 129, 132–135, 147–171] organized by modeling group. The search process revealed a wide range of populations explored. KRI represents the only modeling group that developed and applied a global model, which relates to its focus on global policy. As shown in Table 3, multiple groups modeled the same countries, particularly the polio-endemic countries as of 2006 (i.e. India, Nigeria, Pakistan, and Afghanistan). For each entry, Table 3 shows the population size or time series of population size modeled (N) and the R_0_ used when reported (i.e. entries missing this information did not report it). Values of R_0_ depend on the population, model structure, and poliovirus serotype, so comparisons between different modeling groups for a given population should consider the different attributes of the models identified in Table 2.

**Table 3.** Populations modeled in dynamic transmission models in 83 papers by group, showing population size (N, in millions (M)) (for the time or time series used) and basic reproduction number (R_0_), if reported.

Population	KRI	IC	IDM	Other
**Global**	N = 6,826–8,072 M (2010–2029), R_0_ = 4–13 by WBIL [18, 20] N = 2,526–9,640 M (1950–2100), R_0_ = 4–13 by WBIL for WPV1, WPV1*0.9 for WPV2, WPV1*0.75 for WPV3 [4, 50–62, 65, 77, 81]			
Country group*				
104 GPEI countries	N = 3,600 M in 1988, R_0_ = 7.5 (LI), 9.5 (LMI), 11.5 (UMI) [25]			
Low-income countries	N = 2,933–3,992 M (2010–2029), R_0_ = 10 or 13 [19, 22]			
16 African countries				R_0_ = 1.2–3 for cVDPV2 [133]
European countries				[164]
Importation countries				N = 613 M (2013), R_0_ = V [157]
**GPEI polio-endemic countries (as of 2006)**				
India (Uttar Pradesh and Bihar)	N = 247 M (2006), R_0_ = 16 [19] N = 54–224 M (1950–2100), R_0_ = 13 [33] N = 55–224 M (1950–2100), R_0_ = 13 [35, 36, 46]		[134]	
Nigeria	N = 9.7–186 M (northwest zone, 1950–2100), R_0_ = 8 [33] N = 9.7–234 M (northwest zone, 1950–2100), R_0_ = 7.5 [35, 40, 46, 49, 64]	N = 0.01 M, R_0_ = 5 [100]	[133] N = 0.3 M [129] N = 1.8 M (Kano, 2016), R_0_ = V [132]	
Pakistan and/or Afghanistan	N = 45–422 M (1950–2100), R_0_ = 11 [71, 73–76]			
**Other countries modeled by at least one GPEI-supported modeling group**				
Israel	N = 1.3–15 M (1950–2100), R_0_ = 5–6 [43, 46, 47]			N = 0.050–0.067 M (2012–2014), R_0_ = 1–10 [162] N = 100%, R_0_ = 1.62 [166]
Tajikistan	N = 1.5–11 M (1950–2100), R_0_ = 7–8 [33] N = 1.5–21 M (1950–2100), R_0_ = 8 [35, 46]	N = 5.6 M, R_0_ = 2.16–2.46 [98] N = 5.6 M, R_0_ = 2.58 [99]		
United States of America	N = 145–570 M (1950–2100), R_0_ = 6 [9] N = 318–346 M (2010–2020), R_0_ = 6 [26] N = 158–478 M (1950–2100), R_0_ = 5 [33] N = 0.276 M (2013 Amish), R_0_ = 5 [41] N = 158–462 M (1950–2100), R_0_ = 5 [47]		Houston, Louisiana [134]	N = 0.05–0.09 (deployed military personnel 2015–2025), R_0_ = V [163] R_0_ = V [158]
**Other countries modeled**				
Albania	N = 3.2 M (1996), R_0_ = 11 [10] N = 1.2–1.8 M (1950–2100), R_0_ = 11 [33]			
Bangladesh (Matlab)			N = 0.13 M (2012) [135]	
Cuba	N = 5.9–7.0 M (1950–2100), R_0_ = 8 [33]			
Dominican Republic	N = 3.6 M (2000), R_0_ = 11 [10]			
Haiti	N = 3.2–14.6 M (1950–2100), R_0_ = 9.5 [33]			
Indonesia (Madura Island)	N = 74–254 M (1950–2100), R_0_ = 9 [33]			
Lebanon				N = 7 M (2015) [159]
Mexico (Campo Grande, Capoluca, Tuxpanguillo)				R_0_ = V [170]
The Netherlands	N = 15.2 M (1996), R_0_ = 5 [10] N = 10–17 M (1950–2100), R_0_ = 4 [33] N = 10–16 M (1950–2100), R_0_ = 5 [47]			
Republic of the Congo		N = 2.8 M, R_0_ = 1.5–1.85 [98]		
**Theoretical or hypothetical populations**	N = 100 M, R_0_ = 4–13 by WBIL [10] N = 10 or 100 M, R_0_ = 6–13 [14] N = 0.1 M, R_0_ = 13 [24] N = 0.1–1 M, R_0_ = 8–16 [27] N = 1 M, R_0_ = 10 [34, 69] N = 1 M, R_0_ = 3.6–11.7 [38, 39] N = 0.0035–0.01 M, R_0_ = 15,20,25 [70]	N = 1 M, R_0_ = 3,10 [97]		R_0_ = V [147, 148, 151, 152, 156] N = 100%, R_0_ = 2.8 [149] N = 100%, R_0_ = 4–20 [154] N = 100%, R_0_ = 14 [160, 171] N = 100% (DEB), 0.001–0.1 M (IB), R_0_ = V [155] N = 100 M, R_0_ = 6 [150] N = 100 M, R_0_ = V [153] N = 0.2 M, R_0_ = 8–16 [161] N = 1 M, R_0_ = V [165] N = 0.0035–0.01 M, R_0_ = 15,20,25 [167–169]

* See source for list of included countries.

Abbreviations: cVDPV(1,2,3), circulating vaccine-derived poliovirus(serotype 1, 2, or 3); DEB, differential-equation based; IB, individual-based; GPEI, Global Polio Eradication Initiative; IC, Imperial College; IDM, Institute for Disease Modeling; IPV, inactivated poliovirus vaccine; KRI, Kid Risk, Inc.; LI, low-income countries; LMI, lower middle-income countries; M, million; N, population; R_0_ basic reproduction number; UMI, upper middle-income countries; V = varied (used for R_0_ values, see paper); WBIL, World Bank Income Level; WPV(1,2,3), wild poliovirus(serotype 1, 2, or 3).

## 4. Summary of publications reviewed

This section first describes the polio modeling-related papers from the three groups that support the GPEI according to the timing of their first publication: KRI (starting in 2003), IC (starting in 2006), and IDM (starting in 2014). As discussed in the next three sections, KRI, IC, and IDM each established primary collaborations with three of the GPEI partners, but all three groups benefited from access to GPEI data under a sharing agreement established in 2013 and all groups received financial and/or subject matter expertise support from multiple GPEI partners. Following the detailed discussion of this work, this section provides brief context about the other studies identified in the review that reported on poliovirus transmission modeling or economic analyses related to the polio endgame.

### 4.1. KRI

Motivated by an interest in appropriately integrating economic, risk, decision, and dynamic disease models to demonstrate the difference between static and dynamic policy models and the importance of changes that occur over time, KRI polio modeling efforts began in 2001 [17] with retrospective characterization of the economic benefits of polio risk management in the United States [9]. Informal discussions of the preliminary work on this topic in late 2001 with the US Centers for Disease Control and Prevention (CDC) led to the establishment of a collaboration between KRI and CDC polio subject matter experts [17]. The KRI-CDC collaboration focused throughout the rest of the decade on the polio endgame (i.e. characterization of risks and risk management options for after WPV eradication). In 2003, KRI presented the decision options for post-WPV eradication policies [8] and developed a DEB dynamic transmission model for polio that included immunity states associated with WPV infection and vaccination with OPV and/or IPV, including transmission by individuals with asymptomatic infections [10]. Given the exclusive use of tOPV at that time, this transmission model used a generic poliovirus serotype and did not consider OPV evolution endogenously [10]. KRI focused on the global policy level and developed estimates of the costs for the different post-WPV-eradication decision options stratified by World Bank income levels (WBILs) to capture some important differences that exist between countries [11]. KRI also characterized the costs and value of the information from the global poliovirus laboratory network (GPLN), which supports global poliovirus surveillance [12]. KRI provided the first quantitative risk estimates for VAPP, cVDPVs, and iVDPVs [13]. The risk estimates appropriately varied by WBIL and type of poliovirus vaccine used by national immunization programs based on statistical analyses of available data at the time and as a function of different post-WPV eradication policies [13]. KRI used the transmission model [10] to explore post-WPV eradication outbreak response policies and provided key insights to the GPEI in 2005 [190] about the benefits of both pre- and post-WPV eradication outbreak response [14], which motivated investments in improvements in GPEI outbreak response activities. Many of these papers appeared in a 2006 special issue of *Risk Analysis* [15], which also included perspectives on risk management in a polio-free world [16] and on the history and nature of the collaborative modeling process used [17]. The retrospective economic analysis for the US showed significant (hundreds of billions of 2002 US dollars US$2002) in net benefits from US investments in polio immunization [9], which helped to strengthen US commitments to global polio eradication and risk management.

Following the development of the integrated model components (i.e. dynamic disease transmission, risk, decision, and economic), KRI performed an economic analysis of post-WPV eradication immunization policies [18]. Given the time horizons considered in the economic analyses that extended beyond the characterization of outbreak events, the integrated model included consideration of potential reinfection and asymptomatic participation in transmission of individuals with waned immunity, with paralysis only occurring in a small fraction of fully susceptible individuals. High-level policy discussions related to control vs. eradication in late 2006 motivated KRI to apply the post-eradication model to estimate the economics of eradication (followed by several different post-WPV eradication immunization policies) compared to a wide range of control options [19]. This analysis demonstrated that eradication (if technically and operationally feasible in a reasonable time) represented a better health and economic option than control with OPV in OPV-using countries [19]. Some discussions at the time included significant pessimism about the ability to stop poliovirus transmission in India and the other remaining endemic countries [191]. KRI modeling suggested that elimination could occur in India with sufficient immunization intensity [19] and demonstrated that achieving eradication is a choice (i.e. the actions that countries and the GPEI take matter with respect to outcomes, and neither failure nor success could be taken as a given). KRI also demonstrated the economic inefficiency of a wavering global commitment to eradication [19]. The economic analysis of post-WPV eradication immunization policies showed that either stopping OPV altogether or switching to IPV dominated the continued OPV use (i.e. control) after successful eradication of WPVs [18]. However, using IPV after WPV eradication represented the option with the highest expected costs and the lowest expected cases, while stopping poliovirus immunization represented an option with lower expected costs and some additional expected cases, which led KRI to recommend research and investment into strategies to reduce IPV costs [18]. KRI performed extensive uncertainty and sensitivity analyses [20]. Recognizing the importance of OPV cessation as an option, KRI demonstrated the need for globally coordinated coordination of OPV cessation due to game-theoretic considerations associated with cVDPV risks that could occur with uncoordinated OPV cessation [21]. This analysis also highlighted the importance of creating a stockpile for post-WPV eradication outbreak response [21]. Due to the complexity and scale of the GPEI, KRI recognized the importance of managing the GPEI as a major project and ensuring sufficient resources for polio eradication to succeed [22]. KRI discussions about this work with GPEI partners highlighted the importance of the GPEI taking the long view and asking for the funds that it needed to succeed with a long-term budget and plan, instead of what it thought it could raise in annual budgeting cycles. Although not specific to polio, by extending a simple integrated theoretical model [192], KRI discussed uncertainty and sensitivity analyses for integrated models [193] and explored the dynamics of priority shifting for eradicable diseases [194], the latter of which also built on prior KRI analysis of a wavering commitment to eradication [19]. Recognizing the importance of a stockpile of OPV for post-WPV eradication outbreak response [21], KRI developed a framework for optimal stockpile design [23]. Although KRI primarily used DEB models, KRI developed an IB polio dynamic disease transmission model that showed the significance of different assumptions about mixing networks, which remain highly uncertain and difficult to model at the global level [24].

In 2010, KRI performed an economic analysis that estimated 40-50 billion US$2013 in net benefits for the GPEI for 1988-2035. The range of estimates depended on whether successfully coordinated OPV cessation following WPV eradication included global use of IPV or not (with the lower end of the range of net benefits (i.e. less desirable) reflecting the use of IPV) [25]. That analysis assumed successful WPV eradication in 2012 and considered the impacts of a delay out to 2015 [25]. KRI contributed to discussions about the role of economic analyses in the evaluation of global disease management efforts [195] and the development of eradication investment cases [196], in book chapters not captured by the systematic review. In 2012, KRI explored trends in the risks of poliovirus transmission in the US and recognized that imported live polioviruses could potentially circulate in a population with high IPV coverage, although the risks in the US appeared low [26]. KRI also explored the probability of undetected wild poliovirus circulation after apparent global interruption of transmission [27] (by extending a simple SC model [197] developed and applied in the mid-1990s to support certification of elimination of polioviruses in the Americas [197–199]).

Still focused on post-WPV eradication and the polio endgame, as the GPEI immunization policies evolved, KRI appreciated the need to expand and update its integrated model. Specifically, as the GPEI began using mOPVs, first mOPV1 and then mOPV3, and later using bOPV (which contains both serotypes 1 and 3) for some SIAs, KRI needed to model the transmission of each serotype. KRI identified the need to model population immunity to transmission [1], and widely discussed its key role in prevention [200]. As part of its model update, KRI characterized the global immunization policy options as of 2012 and identified prerequisites for OPV cessation [28]. KRI developed a series of papers published in a 2013 special issue of *Risk Analysis* that described the components of its expanded and updated poliovirus transmission and OPV evolution model and discussed the role of modeling as part of the polio legacy [2]. KRI performed a comprehensive expert review of the literature on poliovirus immunity and transmission [29] and synthesized the information from the experts to (i) numerically characterize an expanded set of immunity states for its transmission model and (ii) identify significant uncertainties despite the large literature [30]. KRI reviewed the 2012 national polio immunization strategies to characterize updated prospective polio immunization policies and reviewed the seroconversion literature to characterize variability in vaccine take rates for different vaccines and numbers of doses in different settings [31]. KRI also updated its prior review of risks [13] and reviewed the literature related to understanding and modeling OPV evolution [32]. Based on this analysis [32], KRI concluded that its prior statistical model for cVDPV risks based on the historical global use of tOPV [13] offered poor predictive value of risks after the GPEI introduced mOPVs and bOPV. Specifically, the poor performance of the statistical model based on historical data [13] when compared with evidence at the time motivated KRI to include OPV evolution and the development of cVDPVs endogenously in its expanded poliovirus transmission and OPV evolution model (i.e. to use a dynamic and serotype-specific approach) [33]. KRI focused on the need to manage population immunity to transmission considering all individuals in the population, including individuals immune to disease but able to contribute asymptomatically to transmission, most notably those with only IPV-induced immunity [34]. The expanded model of poliovirus transmission and OPV evolution offered insights from modeling a diverse set of actual experiences with wild and vaccine-related polioviruses [33]. Overall, the expanded poliovirus transmission and OPV evolution model (i) uses eight recent immunity states to reflect immunity derived from maternal antibodies in infants, only IPV vaccination, only LPV infection, or both IPV vaccination and LPV infection (to more realistically capture the differences in immunity derived from IPV and LPV), (ii) includes multi-stage waning and infection processes (for more realistic characterization of these processes), (iii) characterizes OPV evolution as a 20-stage process from Sabin OPV (as administered) to fully reverted polioviruses with assumed identical properties to typical homotypic WPVs (to allow cVDPV emergence to occur within the model), (iv) characterizes each serotype separately (to analyze serotype-specific poliovirus properties, vaccination policies and risks), (v) considers explicitly both fecal-oral and oropharyngeal transmission (to account for the differential impact of IPV on fecal and oropharyngeal excretion), (vi) accounts for heterogeneous preferential mixing between mixing age groups and subpopulations, and (vii) accounts for differences between various IPV and OPV routine immunization schedules and the reality of repeatedly missed children during successive SIAs [33, 35, 36]. KRI also updated its estimates of IPV costs in the context of exploring national choices related to IPV use with various delivery options [37] and noted continued high expected costs of IPV.

KRI used the updated and expanded integrated global model to identify optimal strategies from a modeling perspective (i.e. with respect to expected health and economic outcomes) to support the GPEI partners as they worked to implement the GPEI 2013–2018 Strategic Plan [201]. In 2014, KRI modeled the dynamics of coordinated cessation of serotype 2 OPV (OPV2) without [38] and with [39] IPV, which demonstrated the importance of using sufficient amounts of tOPV in the run up to OPV2 cessation to increase population immunity to transmission prior to OPV2 cessation [38]. Despite the GPEI emphasis on IPV introduction, these analyses also demonstrated the relatively small expected role of IPV in stopping or preventing transmission in areas with conditions conducive to poliovirus transmission (i.e. relatively high R_0_, high contribution of fecal-oral transmission, like the countries of interest to the GPEI) [39].

Given delays in achieving eradication and requests from the GPEI partners, starting in 2013 KRI began modeling pre-eradication activities and to explore options to help accelerate eradication. KRI applied its transmission model [33] to characterize the potential impact of expanding target age groups for polio SIAs [35] and to stop and prevent poliovirus transmission in two high-risk areas in northern India [36] and in the high-risk area of northwest Nigeria [40]. Considering potential US risks, KRI developed and applied an IB model to characterize the potential for transmission of polioviruses following an introduction of a LPV into the Amish communities in North America [41]. Consistent with prior recognition of the potential for circulation of imported LPVs in areas with high IPV-only coverage based on its US modeling [26], KRI modeled population immunity to transmission and management strategies for Israel following the observation of WPV serotype 1 transmission in Israel despite its high coverage with IPV only [43]. In contrast to some other areas in the US, KRI reported relatively little heterogeneity in six counties in Central Florida at high risk of importations due to international family entertainment attractions [44]. KRI discussed some lessons from the GPEI relevant to measles and rubella eradication [45]. Insights from KRI modeling showed the importance of focusing on immunization program performance (i.e. achieving high coverage with OPV) to maintain population immunity to transmission as the key to success in the polio endgame [42]. Many KRI modeling studies emphasized the failure to vaccinate with OPV as the primary cause of delay in achieving and maintaining WPV eradication, and the importance of heterogeneity in populations that leads to pockets of preferentially-mixing under-immunized individuals that can sustain transmission [35, 36, 40, 41, 43]. KRI provided a high-level review of the policy impacts of its modeling [3].

In 2015, KRI explored the information from different types of poliovirus surveillance activities and modeled the potential for undetected live poliovirus circulation after apparent interruption of transmission [46] based on earlier exploration [27]. KRI characterized global importations and cVDPVs since 2000 and showed that over 50 countries failed to maintain sufficient population immunity to transmission to prevent paralytic cases from cVDPVs and/or imported WPVs [47]. KRI also modeled three countries that use IPV-only for routine immunization (the US, the Netherlands, and Israel) and demonstrated the decline in population immunity in transmission that occurs when countries switch from using OPV to using IPV only. At the time of global introduction of IPV beginning in OPV-using countries, KRI discussed the safety of IPV and emphasized the potential benefits of using IPV as a first dose to reduce VAPP using data from the US experience [48]. Looking closely at northwest Nigeria, KRI explored the trade-offs associated with different strategies to manage population immunity to transmission that demonstrated the high importance of using more tOPV in SIAs in the run-up to OPV2 cessation and the minimal impact of IPV [49].

KRI published a series of articles in a special issue of *BMC Infectious Diseases* in 2015 using its updated integrated model that aimed to help national, regional, and global health leaders navigate the polio endgame from 2013 to 2052. Modeling the long-term risks requires characterization of the potential for reintroductions of iVDPVs from a small number of individuals with B-cell-related primary immunodeficiencies [50], for which KRI reviewed the evidence collected since its 2006 statistical analysis [13]. KRI recognized that static modeling of historical data offered low predictive power for future iVDPV risks. As a result, KRI developed a DES model to support the stochastic generation of iVDPV excreters for prospective risk analyses and the exploration of the potential benefits of polio antiviral drugs (PAVDs) [50]. KRI used its iVDPV model and other stochastic risks related to containment in its integrated global model to characterize the risks, costs, and benefits of different future poliovirus risk management options for 2013–2052 compared to the 2013 baseline, which included continued widespread use of OPV for control [51]. Using both the global model [51] and a model of northern Nigeria [49], KRI showed the importance of vaccine choice and preferential use of tOPV in the run-up to globally coordinated cessation of serotype 2 OPV (i.e. OPV2 cessation), which was then-planned and since implemented in late April 2016 [52]. Recognizing the importance of significant tOPV use and sensitive to the time delays and costs of vaccine production, KRI estimated potential tOPV and bOPV needs through 2020 [53]. As global health policymakers approached the final decision point for establishing the timing of OPV2 cessation, KRI explored alternative OPV cessation and IPV introduction timing options [54] that showed substantial financial benefits associated with delayed IPV introduction. KRI demonstrated the importance of using aggressive and high-quality (i.e. rapid, high coverage, sufficiently large scope) outbreak response SIAs after OPV cessation and during the polio endgame [55]. In anticipation of coordinated OPV2 cessation, KRI explored the risks of potential non-synchronous OPV2 cessation [56] and of inadvertent tOPV use after OPV2 cessation [57]. Later work showed the potential risks of non-synchronous bOPV cessation and inadvertent use of serotype 1 or 3 OPV use after bivalent OPV cessation [58].

Using the updated integrated model [51], KRI performed an uncertainty and sensitivity analysis of cost assumptions [59] that continued to demonstrate the relatively high cost of IPV. Recognizing the importance of maintaining high population immunity for serotypes 1 and 3 prior for future coordinated bOPV cessation, KRI demonstrated the benefits of high levels of continued bOPV use and sustaining OPV production through bOPV cessation [60]. Building on prior characterization of iVDPV risks [50], KRI modeled the impact of comprehensive screening to find and treat asymptomatic iVDPV excretors and explored the impact of screening on the expected benefits of PAVDs [61]. KRI explored the potential benefits of investments in a new, ideal poliovirus vaccine assuming the best attributes of OPV and IPV [62]. Emphasizing the importance of actions taken by countries and the GPEI, KRI highlighted the importance of maintaining preparedness throughout the polio endgame [63]. KRI also demonstrated the minor role of IPV in outbreak response when used in conjunction with OPV, and showed that IPV in addition to OPV for outbreak response (in the outbreak area) does not represent a cost-effective option compared to using OPV alone [64]. KRI demonstrated the need to maintain sufficient poliovirus vaccine supplies and stockpiles for outbreak response in the polio endgame [65] and assessed the economic benefits of temporary recommendations for international travel immunization requirements for countries with transmission of WPV1 [66].

Recognizing the increasing role of environmental surveillance for polioviruses, KRI systematically reviewed published poliovirus environmental surveillance studies and reported information related to the design, cost, and effectiveness of these systems [67]. KRI also explored the dynamics of die-out of serotype 2 polioviruses after homotypic OPV cessation and lessons learned from its cessation relevant to the cessation of OPV serotypes 1 and 3 [68]. Reviewing insights from prior modeling [35, 36, 40, 41, 43], KRI demonstrated how under-vaccinated subpopulations can sustain poliovirus transmission despite high coverage in the surrounding population, depending on the degree of mixing and the size of the under-vaccinated subpopulation [69]. Building on these lessons, KRI explored the potential for silent circulation of live polioviruses in small populations [70], and the role of hard-to-reach subpopulations in characterizing the confidence about the absence of transmission for purposes of certifying the eradication of WPV1 [71]. KRI revisited its earlier characterizations of containment risks [13, 51] and explored current containment risks and their management [72]. KRI also discussed the role of system dynamics in integrated polio risk management modeling [4].

With continued failure to stop transmission in Pakistan and Afghanistan as of 2016, KRI developed a model of both countries as one epidemiologically connected area [73]. Modeling poliovirus transmission in Pakistan and Afghanistan suggested that subpopulations of under-vaccinated individuals that preferentially mix with each other probably sustain transmission and that interrupting transmission requires a significant improvement in OPV SIA coverage in these under-vaccinated subpopulations [73]. Further modeling of poliovirus transmission in Pakistan and Afghanistan suggested the need for proactive strategies (as opposed to reactive ones) to stop poliovirus transmission [74], and KRI cautioned against getting distracted by the introduction of IPV from achieving high coverage with OPV SIAs. Exploration of the potential for silent poliovirus transmission in Pakistan and Afghanistan [75] showed the role of surveillance in providing confidence about the absence of transmission. Tradeoffs in key characteristics of the poliovirus surveillance system in Pakistan and Afghanistan [76] suggest some role of environmental surveillance in assuring confidence about the absence of transmission, although KRI identified the need for further characterization of the quality of the information from polio surveillance in Pakistan and Afghanistan to fully explore the benefits of investments in environmental surveillance.

Looking prospectively at the polio endgame given failure to succeed in the GPEI objectives by 2018, KRI discussed the role of different poliovirus risks and risk management opportunities [72], and the potential risks of needing to restart OPV [77]. KRI also reflected on the role of integrated modeling to support the global eradication of vaccine-preventable diseases [4].

In 2019, KRI updated its cost estimates of the GPLN including both acute flaccid paralysis (AFP) and environmental surveillance [78]. KRI characterized the impact of hard-to-reach subpopulations on confidence about no undetected circulation in the context of supporting global certification of wild polioviruses [71]. Building on prior recognition of the potential role of a new vaccine [62], KRI commented on an article that reported the results of a new OPV2 vaccine strain (nOPV2) [79] and explored the logistical challenges of modeling and implementing a restart of OPV after its cessation [80].

Although outside of the time window for this review, in early 2020, KRI published an updated version of its integrated model to account for the programmatic experience, vaccination achieved, and epidemiology through 2019 [202]. This process included updating the inputs for its iVDPV risk model [81], and focused on actual and expected performance throughout the polio endgame instead of assuming optimistic and ideal risk management from 2015 on [203] as KRI assumed earlier [51].

### 4.2. IC

Starting in 2006, IC began reporting on its application of advanced epidemiological methods to support the GPEI as part of its collaboration with the World Health Organization (WHO). IC focused on statistical analyses of existing data and data collected as part of prospective clinical trials or challenge studies and did not perform any economic analyses. With respect to transmission modeling, between 2000 and 2019, IC applied dynamic transmission models to explore several specific topics. In 2013, using a simple DEB and SC model on two hypothetical populations, IC explored IPV use after OPV cessation, which suggested that IPV would protect children from paralysis, and under some conditions, IPV use could potentially limit transmission [97]. The study also noted that IPV use in routine immunization could also potentially delay the detection of outbreaks and allow transmission to spread further by preventing AFP cases [97]. In 2014, IC used an SC model to explore the impact of older age groups on the transmission of polioviruses, which identified faster outbreak response as substantially more important than expanding the age range of campaigns [98]. IC applied the same SC model in 2017 to explore a statistical inference framework to epidemiological and genetic data collected during a poliovirus outbreak to estimate transmission parameters [99]. Using an SC model for Nigeria, in 2016 IC characterized the role of tOPV SIAs before OPV2 cessation and suggested that in closed populations with no routine immunization coverage, conducting tOPV SIAs with some characteristics (e.g. one SIA with low coverage) could increase cVDPV2 risks after OPV2 cessation [100]. The inclusion of low routine immunization coverage in the model suggested the need for a sufficient number of focused tOPV SIAs before OPV2 cessation in areas at risk of VDPV2 emergence to raise population immunity above the transmission threshold [100].

IC also used statistical models to characterize transmission dynamics. Using data from Nigerian nonpolio AFP cases, IC applied a Poisson mixed effects model to characterize the connections between local government areas (LGAs) and suggested that a radiation model of human mobility provided the best fit [101]. IC applied a similar model to data from Pakistan and found that movement dynamics did not provide strong predictors for future cases and highlighted the necessity of improved SIA quality [102].

IC performed multiple case–control studies that estimated the efficacy of poliovirus vaccines using nonpolio AFP surveillance data collected by the GPLN, many of which supported GPEI decisions to introduce additional poliovirus vaccine formulations (e.g. mOPV1, bOPV, IPV) as new tools that would accelerate eradication. The first case–control study published by IC estimated the efficacy of tOPV vaccine in India, with a focus on areas with high population density and poor sanitation (i.e. Uttar Pradesh and Bihar) in which poliovirus transmission remained endemic [82]. This analysis showed poor tOPV efficacy per dose in these areas and suggested that using some mOPV1 SIAs in these areas could help to stop WPV1 transmission without significantly increasing WPV3 risks [82]. Subsequent case–control studies estimated vaccine efficacy of mOPV1 on the order of three times higher for serotype 1 poliomyelitis disease than for tOPV for Uttar Pradesh and Bihar [83] and for polio-endemic areas in northwest Nigeria [84]. Building on this work, IC led a challenge study in northern India to assess mucosal immunity induced by OPV, which demonstrated significant differences by location, serotype, vaccine formulation, and the number of doses [85]. IC assessed rates of excretion of live polioviruses (wild and OPV-related) in asymptomatic children in contact with suspected cases as a function of age, OPV doses received, and characteristics of the suspected case, which confirmed some asymptomatic participation in WPV transmission by OPV-vaccinated children [86]. Following the introduction of mOPV1 and mOPV3 in SIAs in Nigeria, IC compared the clinical characteristics of reported polio cases, estimated vaccine efficacy for different OPV vaccine formulations, and highlighted the improvements in vaccine-induced immunity against serotypes 1 and 3 and the decline in immunity to serotype 2 in children 0–2 years of age, which resulted in increased observations of cases caused by cVDPV2s [87]. IC explored the duration of mucosal immunity induced by OPV in India and suggested that it wanes significantly within 1 year [88].

Following the introduction of bOPV, in 2012, IC performed a case–control study using data from young children in Pakistan and Afghanistan that reported comparable effectiveness of bOPV to mOPV1 for serotype 1 and commented on the poor and declining immunization coverage in these countries [89]. In 2014, IC reported on the results of trials in India that demonstrated that the delivery of a supplemental IPV dose to previously-OPV-vaccinated children <5 years old boosted their intestinal immunity [90], and does so more effectively than a supplemental OPV dose [91]. Following this study cohort, in 2017 IC reported that the duration of boosting by IPV of intestinal immunity in OPV-vaccinated children remained elevated for 6 and 11 months, but showed evidence of waning [103]. Using data from Nigeria, in 2014 IC explored the vaccine effectiveness for the different formulations of OPVs in use (i.e. mOPVs, bOPV, tOPV) and suggested that immunity in children <3 years old to serotypes 1 and 3 had improved with the use of mOPVs and bOPV [92]. In 2016, using data from Indian infants 5–11 months old, IC reported that the number of tOPV doses received represented the main determinant of serotype 3 seropositivity [104], and reported results from a clinical trial that suggested that a 3-day course of azithromycin prior to delivery did not improve the immunogenicity of mOPV3 [105]. In 2018–19, using this same population, IC reported findings that showed a correlation between the quantity of virus shed and the magnitude of the serum neutralizing antibody response at 21 or 28 days [106], showed a greater impact on OPV response by enteric viruses than bacterial microbiota [107], and that did not show an association between seroconversion from one dose of mOPV3 and FUT2 genotype (i.e. single-nucleotide polymorphisms G428A, C302 T, and A385 T) [108].

In addition to analyzing results from clinical trials and challenge studies, IC also developed statistical models to characterize risks and effectiveness of some interventions by analyzing available data. In 2011, to explore the widespread transmission of WPVs in Africa, IC applied a statistical model that identified the proximity to the continued transmission in Nigeria and poor performance of national immunization programs in some neighboring countries as risk factors for transmission of reintroduced WPVs in Africa [93]. In 2017, IC revisited this topic for both Africa and Asia, concluded that low population immunity represented a key risk factor for WPV or cVDPV transmission, and recommended maintenance or improvement of vaccination in the high-risk areas it identified [109]. In 2015, IC applied a statistical model to estimate the effectiveness of SIAs using nonpolio AFP cases reported for children <2 years old in Pakistan, which showed temporal changes in coverage and identified some under-vaccinated populations [110]. Building on this work, in 2016 IC characterized spatial and temporal trends in vaccine-induced population immunity for serotype 2 for Nigeria and Pakistan prior to OPV2 cessation to explore the need for additional serotype 2-containing vaccines [111]. In 2016, using retrospective surveillance data, IC suggested that developing a real-time database of notified AFP cases and applying a Poisson space-time scan statistic at weekly intervals could potentially lead to earlier outbreak response [112]. In 2017, a year after OPV2 cessation IC analyzed the surveillance data and concluded that high population immunity prior to OPV2 cessation facilitated the die out of serotype 2 OPV-related viruses in most areas, but that cVDPV2 circulation continued in areas at high risk for transmission [113]. IC also performed a statistical analysis that explored the impacts of using IPV in addition to OPV for outbreak response in Pakistan and Nigeria and suggested some benefit of using IPV although the results were not statistically significant [114] and an updated analysis for Pakistan in 2018 [115]. In 2018, IC analyzed different sources of routine immunization data in Pakistan that showed both variable data quality and heterogeneous coverage [116] and assessed the sensitivity of poliovirus surveillance (both AFP and ES) for serotype 1 [117].

Between 2000 and 2019, IC also contributed a number of reviews to the literature. Recognizing the wealth of studies published over decades, IC systematically reviewed the OPV challenge studies that evaluated the induction of immunity from OPV and/or IPV against shedding, which concluded that immunization with IPV would likely show limited impact on poliovirus transmission in countries characterized by fecal-oral poliovirus transmission [94]. IC discussed some of the challenges for the polio endgame with a focus on issues related to OPV vaccine failure [95], results of clinical trials performed by others that added IPV to routine immunization schedules in OPV-only using countries [118, 119] including potential impacts of IPV on mucosal immunity [120], and showing no benefits of adding IPV in mOPV2 outbreak response SIAs [121]. IC also commented on biological challenges that limit the effectiveness of vaccines in the developing world, including OPV [122], and the need for innovation in poliovirus surveillance, vaccines, and vaccination strategies [123]. IC also systematically reviewed IPV vaccine effectiveness studies [96] and the impact of IPV on mucosal immunity [124], and suggested that IPV use could play a key role in halting poliovirus transmission and hasten polio eradication due to boosting of immunity of individuals previously given OPV [124]. IC also systematically reviewed the characteristics of known iVDPVs [125], interventions to improve oral vaccine performance [126], and the effect of different vaccine schedules on humoral and intestinal immunity against poliovirus [127].

### 4.3. IDM

IDM, an institute within the Global Good Fund, is a collaboration between Intellectual Ventures and Bill and Melinda Gates. IDM established a GPEI-partner collaboration with the Bill & Melinda Gates Foundation in 2011. IDM published its first polio model-related work in 2014 in a review of poliovirus infection and immunity, which it discussed in the context of developing inputs for use in an individual-based model [128]. Using an IB mathematical model, IDM explored the use of expanded age groups in SIAs and concluded that these would not significantly improve the prospects of achieving polio eradication [129]. In 2016, IDM used an IB model of children <5 years old in Kano, Nigeria, which suggested a high probability of elimination of transmission of WPV1 from Kano as of October 2015 [132]. In 2017, IDM applied an IB model of a hypothetical cVDPV2 outbreak response in northwest Nigeria, which suggested that the use of mOPV2 for outbreak response could seed new cVDPV2 lineages as early as 18 months after OPV2 cessation [133]. This analysis discussed the importance of rapid and aggressive outbreak response and the potential role of IPV, including the possibility of its use delaying detection of an outbreak [133]. In 2018, IDM described another IB model in detail and demonstrated the ability of the model to reproduce historical outbreaks in different transmission settings based on historical data [134]. IDM used this extensive and well-documented IB model to explore the stability of polio eradication after the withdrawal of OPV [134]. This analysis highlighted the fragility of eradication and the importance of strategies to stop any post-cessation outbreaks and the potential need for new vaccine tools, while suggesting a limited role for IPV in high transmission settings [134]. Building on this work, IDM used the results of a field trial in Bangladesh designed to collect fecal shedding data after mOPV2 challenge and this IB model to explore community transmission of OPV2-related viruses after OPV2 cessation, which suggested an increase in transmission risk over time after OPV2 cessation [135].

IDM also performed multiple statistical analyses using GPLN data. In 2014, IDM discussed the use of lot quality assurance sampling (LQAS) to evaluate the quality of SIAs [130] and used Nigerian AFP surveillance data to predict the risks of cases at the district level [131]. In 2015, IDM also developed a simple statistical model of the polio force of infection using data from Nigeria and based on anticipated die out of all wild poliovirus transmission in Nigeria in 2015 [136]. IDM provided a perspective on the application of advanced digital tools (e.g. GIS tracking) to fight polio and other communicable diseases [137]. In 2015, IDM also applied a heuristic algorithm to spatially reconstruct partially observed transmission networks using phylogenetic data for northern Nigeria and found substantial limitations of the method due to under-sampling [138]. Building on this work, in 2016 IDM characterized OPV revision using whole-genome sequencing data from Nigeria, which showed some evidence of transient and local transmission of OPV-related serotype 1 and 3 viruses during periods of low wild polio incidence that appeared consistent with national OPV use [139]. IDM performed a statistical analysis of immunization data to characterize OPV-induced population immunity and assess campaign effectiveness in high-risk countries to support GPEI SIA planning activities [140]. Using data from Nigeria, IDM constructed a hierarchical model to estimate SIA effectiveness to characterize OPV-induced immunity and compared these estimates to data from LQAS and incidence data [141]. Using these methods, in 2017, IDM reported spatial risk model predictions and recommended subnational prioritization to accelerate poliovirus elimination in Pakistan [142]. Following OPV2 cessation, IDM compared pre- and post-cessation detection rates of cVDPV2s and showed the die out of OPV2-related viruses in most countries [143].

In 2018, IDM reviewed its applications of IB modeling for multiple pathogens, including polio [144]. IDM also used data from Pakistan and Afghanistan to assess the sensitivity of poliovirus environmental surveillance [145]. In 2019, IDM reported the results of a cost study that compared polio eradication to indefinite control with 2 doses of IPV and multiple doses of OPV in currently OPV-using countries [146].

### 4.4. Poliovirus transmission modeling studies published by other authors

In 2001, one study used a DEB model to characterize poliovirus transmission as part of an analysis that explored the probability of detecting poliovirus in sewage water as a function of different transmission conditions (e.g. equilibrium and non-equilibrium) [147]. Building on DEB modeling performed and applied prior to 2000 [204, 205], one 2001 study reported the application of a simple DEB model to characterize the expected infections and cumulative infections as a function of time since poliovirus introduction into a naïve population as a function of different net reproduction numbers ([148] see Annex). Although not captured in the review, additional perspectives by the same author published since 2000 addressed challenges for the polio endgame [206, 207], risk factors for the severity of outbreaks after eradication [208], and characterization of the extent of VDPV infections [209].

A 2005 study used a DEB model to characterize WPV in the absence of vaccines, which characterized polio as a disease of development (i.e. a disease that becomes worse as hygienic conditions improve such that individuals become infected at relatively older ages when the symptoms present as more severe) [149]. In 2008, following widespread recognition of cVDPVs, one study applied a DEB model to explore three alternative eradication strategies using pulsed OPV or continuous or pulsed IPV immunization and different levels of coverage [150]. However, this theoretical analysis ignored the benefits of secondary transmission of OPV and the complexity of reinfection and included simple modeling of the reversion of OPV given to vaccine recipients, which the authors refer to as cVDPVs but which behave more like VAPP [150]. A 2010 study by the same authors applied a DEB model that included secondary OPV transmission, which explored continuous and pulsed OPV immunization strategies [151]. A 2011 simple theoretical DEB model assumed that IPV can precipitate paralysis in a patient already incubating a poliovirus infection, and suggested sick and unimmunized children should not receive IPV during polio epidemics [152].

In 2012, a comprehensive theoretical DEB model that included OPV secondary infections, OPV evolution, and IPV use explored the dynamics of OPV cessation and the probability of eradication [153]. A 2013 study applied a DEB model that considered waning immunity and showed how countries with high transmission conditions remain at risk for epidemics from the reintroduction of WPV, which offered some explanation for challenges that prevented successful poliovirus elimination in some countries [154].

In 2015, two independent theoretical studies used DEB models to characterize the dynamics of OPV and cVDPV transmission in populations as a function of coverage and the competition for infectible individuals [155, 156]. One of these studies included an IB version of the model to simulate die out and discussion of the dynamics of small population sizes [155]. Another study in 2015 applied a simple DEB model to highlight the increasing role of reintroduction of polioviruses by travelers [157]. Another study applied an SC model to fit an SIR model to pre-vaccine US incidence data to infer WPV infection dynamics and variable time and space R_0_ estimates [158], which concluded that contrary to a prior study [149], polio does not appear to be a disease of development. Assuming the existence of an environmental reservoir for live polioviruses, one study characterized the impacts of different pulse vaccination strategies in a DEB metapopulation model and highlighted the importance of synchronization [160].

In 2016, one study explored the ability to detect polio cases in populations with high IPV coverage, which highlighted that asymptomatic infections may mask live poliovirus transmission and suggested longer delays to detection as vaccine coverage and/or the proportion of the population with only IPV vaccination increases [210]. Revisiting a simple theoretical model of silent circulation developed in the mid-1990s [197] and reconsidered by KRI in 2012 [27], a 2016 analysis emphasized further limitations of the simple model with respect to consideration of the vaccination history [161]. Modeling the experience with WPV1 reintroduction into Israel, one study used a DEB model to characterize the importance of using OPV to interrupt WPV transmission in a developed country with very high IPV coverage [162]. A theoretical DEB model highlights OPV as an example of a weakly transmissible vaccine for which the transmissibility of the vaccine can help with global eradication efforts [211].

One study in 2017 applied a DEB model to explore the implications of using a deployment-risk-based immunization strategy (i.e. to polio-endemic areas) for US military personnel and nondeployed US military populations [163]. Focusing on the dynamics of a hypothetical importation of WPV1 from Syria into Lebanon in 2013 to explore the potential benefits of an OPV SIA conducted in Lebanon in November 2013, a 2017 study developed an IB model that demonstrated the importance of the preventive SIA with respect to preventing a potentially large and explosive outbreak [159].

Considering the potential impacts of importations of poliovirus into IPV-using countries by large groups of immigrants, a 2017 analysis used a DEB model to explore the vaccination required in both groups to stop transmission [164].

Koopman and colleagues published multiple modeling papers between 2017 and 2019. The first study built on earlier work [154] although the 2017 analysis used a relatively simpler DEB model with much more extensive analysis of waning immunity and suggested potential challenges associated with OPV cessation due to potential silent poliovirus transmission in some areas and the potential role of environmental surveillance [165]. A separate study applied a DEB model to the importation of WPV1 in Israel and emphasized the importance of environmental surveillance [166]. A series of three papers used SC models to explore the potential for undetected transmission in theoretical small and isolated populations [167], the impact of using unrealistically high values for the basic reproduction number that limited generalization of the prior results [168], and an extension of an independent reanalysis [70] of the first paper [167] to include different assumptions about waning [169].

Recently, a 2018 study applied an IB model calibrated to stool shedding data from communities in Mexico to explore the impacts of using OPV for outbreak response 5 years after OPV cessation [170]. In 2019, one theoretical DEB modeling exercise explored the potential role of human exposure to polioviruses from the environment [171].

Although not captured by the systematic search or included in the review, readers may also find other polio models published prior to 2000 of interest. These include a DEB model of an outbreak in Taiwan [212], DEB models to support eradication planning published in 1994 [213] and 1996 [198], and three papers published in 1995–6 related to undetected circulation at the time of certification [197, 199, 214].

### 4.5. Economic analyses published by other authors

The systematic search identified some additional economic analyses, and we include mention of others known to the authors. For example, the search did not find a 2003 study that estimated the costs and benefits of polio eradication by WHO region [215] or a 2004 cost analysis of potential post-eradication polio immunization policies [216], and by design, we missed economic analyses of polio eradication published prior to 2000 [217, 218]. The search included one 2000 analysis that explored pricing for combination vaccines that included IPV in the US [172]. One 2001 study reported that introducing IPV in Australia did not appear cost-effective [173], which reached conclusions similar to 1988 [219, 220] and 1996 [221] studies for the US. The search did not capture other studies that reached similar conclusions for IPV introduction in 2006 for South Africa [222] or in 2008 for OPV-using countries generally [223]. The search also did not find a 2005 study for Mexico [224] or a 2017 study for India [225] that suggested that stopping OPV SIAs and eliminating their costs could potentially off-set the costs of IPV introduction. The search captured two economic analyses published in 2006 that reported decision analysis results comparing vaccine options for responding to a poliovirus outbreak in the US from a vaccine stockpile [174] and comparing pre-vaccination serological testing vs. presumptively vaccinating internationally adopted and immigrant infants in the US [175]. The search also identified an economic analysis that explored the incentives of individual countries to participate in global polio eradication with consideration of post-eradication risks [176], which built on prior related studies by the same author not captured in the search [226, 227]. The search did not include a subsequent 2013 discussion of the multiple economic games occurring in the final stages of polio eradication [228]. The search included a 2014 study that found that switching from 10-dose to 5-dose vials of IPV reduced wastage but did not appear cost-saving for the studied vaccination facilities in Bangladesh, India (Uttar Pradesh), Mozambique, and Uganda [177]. A 2015 review of economic analyses related to disease elimination and eradication initiatives included a number of studies included in the search, but did not appear in the search results [229]. The search identified a 2016 study that estimated the health and economic benefits of three decades of polio elimination investments in India [178]. Finally, the search captured a 2017 study that reported on the GPEI costs associated with supporting tOPV-using countries as they switched to bOPV [179]. The search did not capture a 2019 study that reported the cost per child vaccinated with full versus fractional-dose IPV [230].

## 5. Themes

In the process of extracting data from the different studies, we captured some common themes in Table 4 and we identified instances in which the modeling groups provided similar or conflicting insights or recommendations.

**Table 4.** Summary of themes explored by multiple modeling groups.

Theme	KRI	IC	IDM	Other
Outbreak response speed	[14, 35, 43, 55]	[98]		[162, 166]
Expanded age group SIAs	[35]	[98]	[129]	
Population immunity*	[10, 16, 18, 19, 26, 33–40, 43, 46, 47, 49, 52–58, 60, 68–70, 73–77]	[83, 84, 87, 89, 92, 93, 100–102, 109, 111]	[131, 133, 140–142]	[155, 162]
OPV cessation dynamics	[26, 38, 39, 49, 68]	[100, 111, 113]	[134, 143]	[153, 165, 166]
Silent transmission on an IPV background and/or delayed detection of transmission due to IPV use	[26–28, 39, 41]	[97]	[133]	[162, 164, 166, 170]
Role of IPV after OPV cessation	[18–20, 25, 28, 33, 39, 51–60, 64, 65, 68, 69, 73–77, 80]	[90, 91, 94, 97, 103, 114, 115, 118–120, 122]	[133]	[153]
Undetected circulation	[27, 46, 70, 71, 75, 76]		[132, 136]	[161, 165, 167–169]
Role of IPV in outbreak response SIAs	[51, 55, 64, 68]		[133]	[150, 164]
Environmental surveillance	[43, 46, 55, 67, 71, 73–76, 78]	[117]	[136, 145, 147]	[162, 166]
Vaccine stockpile	[21, 23, 53, 65, 77]			[174]
iVDPVs	[4, 13, 50, 61, 81]	[125]		

Abbreviations: IC, Imperial College; IDM, Institute for Disease Modeling; IPV, inactivated poliovirus vaccine; iVDPVs, immunodeficiency-associated vaccine-derived poliovirus; KRI, Kid Risk, Inc.; OPV, oral poliovirus vaccine; SIAs, supplementary immunization activities.

* As indicated in text, defined differently by the 3 modeling groups: **KRI** focuses on modeling infection and defines ‘population immunity to transmission’ based on all individuals of all ages integrated over all immunity states in a DEB model as a function of serotype, population-specific inputs, and time, which is a model-based concept that does not vary by paper (see details in [34, 202]). KRI publications earlier than 2013 discussed ‘population immunity’ as the same concept (i.e. over the entire population), but characterized it as an input for some analyses based on data (see e.g. [10]); **IC** focuses only paralysis (i.e. not infection) and defines ‘population immunity’ including only vaccine-induced immunity (i.e. excluding immunity from maternal antibodies and immunity induced by infection with any live poliovirus via community spread), and varies by paper depending on the data used (e.g. nonpolio AFP data for: serotype 1 only for children <5 years old [83, 84, 87], serotypes 1 and 3 for children <2 years old [89], serotypes 1, 2, and 3 for children <36 months [92], serotype 1 for children <5 years old [101], serotype 2 for children <2 years [100], serotype 2 for children <36 months [111, 113], and serotype 1 for children <36 months [102]; multiple metrics used for regression analyses [93, 109], see individual papers for specific definitions); **IDM** definition of ‘population immunity’ includes only vaccine-induced immunity (i.e. excluding immunity from maternal antibodies and immunity induced by infection with any live poliovirus via community spread), focuses on paralysis (i.e. not infection), and varies by paper depending on data used (e.g. OPV-induced immunity for nonpolio AFP cases in children <5 years old in a district within a 6-month period [131, 133, 141, 142], children <15 years old [140], dose estimates based on SIAs, see individual papers for specific definitions).

### 5.1. Responding quickly to outbreaks

We found consistency in the recommendations made independently from different transmission modeling studies [14, 35, 43, 55, 98, 133, 162, 166] with respect to the importance of rapidly detecting and responding to outbreaks. Multiple studies also recommended that in the event of detection of a transmitting virulent virus (i.e. WPV or cVDPV) after OPV cessation, using OPV for outbreak response offered the best option [14, 35, 52, 55, 68, 133, 134], although its use comes with risks. Specifically, all three modeling groups expected the risks associated with using OPV for outbreak response after OPV cessation would increase as a function of the time since cessation (i.e. as more birth cohorts without exposure to LPVs accumulate). The modeling motivated the creation of mOPV vaccine stockpiles for outbreak response after OPV cessation to ensure sufficient supplies. For the review inclusion time (2000–2019), only KRI applied transmission modeling to questions related to creating, funding, and managing stockpiles of poliovirus vaccines [21, 23, 53, 65, 77], although one economic analysis considered the US stockpile [174].

### 5.2. SIAs with expanded age groups

All three modeling groups gave similar recommendations to the GPEI partners based on the application of transmission models in response to questions about the potential benefits of using expanded age group as the target for SIAs [35, 98, 129]. Notably, although the populations modeled by the groups differed, the primary conclusions of the application of transmission models to the question of expanding the target age ranges for OPV SIAs emphasized the importance of reaching susceptible children (typically the younger ones and those in undervaccinated subpopulations) as quickly as possible. Some of the modeling groups also highlighted the substantially lower cases (and costs) associated with performing pSIAs to prevent the need for oSIAs [34, 35, 159].

### 5.3. Population immunity

All of the modeling groups recognize the need for high population immunity to achieve and maintain polio eradication. However, one of the most notable sources of conflicting recommendations from the three modeling groups comes from the use of different definitions for population immunity. As shown in Table 4, all three modeling groups used the term ‘population immunity’ in numerous 2000–2019 publications. The KRI papers that mention population immunity use a dynamic transmission model that focuses on the characterization of the transmission of infections based on the understanding that eradication requires achieving and maintaining the end of all LPV transmission (i.e. permanent prevention of infection). As such, KRI defines ‘population immunity to transmission’ for each serotype as dynamic measure of the overall immunity by serotype of all individuals in a population, including partial immunity for those with prior vaccination or infection who can become (re)infected and participate in transmission due to the nature or waning of their immunity. In contrast, statistical and epidemiological models developed by IC defined population immunity differently, even from paper to paper depending on the research question and data used, see note at the bottom of Table 4, which indicates the serotype-specific definitions applied in some papers. The IC concept of population immunity focuses on vaccine coverage and prevention of paralysis (instead of infection). While this narrower concept of population immunity provides an indication of susceptibility to transmission in an important part of the population (i.e. young children) and can characterize variability in relatively small geographic areas (e.g. districts), it excludes the (i) the immunity of young children induced by exposure to WPVs, secondary spread of OPV-related viruses, and cVDPVs, (ii) serotype-specific immunity in some instances (particularly when countries use mOPV or bOPV), (iii) differences in the nature of immunity induced by OPV and IPV, and/or (iv) the potential role of older children and adults in transmission. The IDM papers that discuss population immunity also focus on vaccine coverage in young children. In the review, we noted two other modeling studies that mentioned population immunity [155, 162]. Although not captured by the review, a study of the impact of SIAs in the Democratic Republic of the Congo also estimated population immunity and emphasized the importance of achieving and maintaining high population immunity [231].

### 5.4. OPV2 cessation dynamics

All three modeling groups provided recommendations to the GPEI related to OPV2 cessation. KRI integrated modeling [18] helped to support the GPEI establishment of a 2008 global agreement to stop OPV use after WPV eradication [232], and to do so with globally coordinated OPV cessation and with the contingency of mOPV vaccine stockpiles for outbreak response [21]. Despite delays in achieving WPV eradication, later integrated analyses reaffirmed this strategy [51, 54], while also emphasizing the need to carefully manage the risks associated with OPV cessation and to ensure sufficient OPV vaccine supplies [52, 53]. In preparation for OPV cessation, KRI applied DEB modeling to explore OPV cessation dynamics and recommended that the GPEI partners increase population immunity to transmission for serotype 2 to stop any existing cVDPV2s and prevent the creation of future cVDVP2s prior to globally coordinating OPV2 cessation by intensifying tOPV pSIAs [26, 38, 39, 49, 52–54, 68]. IC used an SC model to explore theoretical concepts related to OPV cessation dynamics [100]. When first presented to the GPEI partners, this modeling initially did not consider the seeding of OPV2 from routine immunization in all tOPV-using countries, which led IC to recommend caution about tOPV pSIAs and contrasted with the recommendations from KRI [38, 39]. However, in its published results, IC considered tOPV use in routine immunization, and supported the strategy of ‘focused tOPV SIAs before OPV2 withdrawal in areas at risk of VDPV2 emergence and in sufficient number to raise population immunity above the threshold permitting VDPV2 circulation’ [100]. A separate statistical analysis by IC supported the GPEI decision to globally coordinate OPV2 cessation in 2016 based on its assessment and expectations about population immunity for Nigeria and Pakistan [111]. After OPV2 cessation, IC and IDM performed statistical analyses that reported that the high population immunity achieved in most areas helped with the prevention of cVDPV2s [113, 143], while also noting problem areas. KRI and IDM characterized the expected increasing vulnerability of populations to transmission of serotype 2 LPVs as a function of time after OPV2 cessation and the risks posed by reintroductions of LPVs from multiple potential sources, including the risks of using mOPV2 use for outbreak response [56–58, 60, 133]. After OPV2 cessation, in a review of lessons learned KRI emphasized the importance of reaching under-vaccinated subpopulations [69], characterized the probabilities of potentially needing to restart OPV2 vaccine production and use on a large scale [77], and discussed the complex vaccine choices and logistics of managing vaccine supplies [80]. Several studies by others also explored the dynamics of OPV cessation and the risks of reestablished transmission [153, 170].

### 5.5. IPV

Numerous studies explored the role of IPV use after OPV cessation [18–20, 25, 28, 33, 39, 51–60, 64, 65, 68, 69, 73–77, 80, 90, 91, 94, 97, 103, 114, 115, 118–120, 122, 133, 150, 153, 164], primarily related to IPV use in routine immunization after WPV eradication. These studies included consideration of the use of IPV in oSIAs, which represents a topic on which the modeling groups offered different recommendations [51, 55, 64, 68, 133, 150, 164]. Notably, KRI does not recommend the use of IPV for oSIAs in OPV-using countries except when homotypic OPV is not available, because adding IPV to oSIAs is not effective and not cost-effective based on its DEB and integrated modeling [64]. In contrast, IC suggests that adding IPV may offer some benefit based on statistical modeling of observational data [106, 114]. The health and economic benefits of using IPV in routine immunization in OPV-using countries differ substantially before and after homotypic OPV cessation. Giving IPV doses sequentially before OPV doses in a national immunization schedule can eliminate VAPP, which is important in high- and upper middle-income countries that achieve high coverage and want to minimize risks associated with vaccine use. In contrast, for countries with relatively lower coverage, IPV may provide some protection from paralysis to the small fraction of children who only receive IPV, but it does not substantially contribute to population immunity to transmission and it may lead to the potential for silent transmission or delayed detection of transmission of LPVs [26–28, 39, 97, 133, 162, 164, 166, 170]. The high cost of IPV also remains an issue, with the relatively high cost of the vaccine and its administration making IPV use not cost-effective. IPV offers an expensive option for post-OPV cessation insurance (i.e. a vaccine that provides protection from paralysis to recipients at a high cost for a virus that is supposed to be gone and does not limit participation in transmission if the virus is not gone or is reintroduced).

### 5.6. Undervaccinated subpopulations and ‘weak links’

All of the modeling groups recognized the role of undervaccinated subpopulations in sustaining LPV transmission and recommended focus on these weak links. However, the groups recommended different strategies. Based on the application of its DEB modeling, KRI repeatedly emphasized the need to overcome the failure to vaccinate these subpopulations and to reach all populations with sufficient quantities of tOPV prior to OPV2 cessation, and bOPV after OPV2 cessation to achieve high levels of population immunity to transmission to stop and prevent WPV and cVDPV transmission [36, 38–40, 42, 47, 49, 68, 69, 73, 74]. In contrast, IC emphasized vaccine failure based on its characterization of low OPV efficacy from case–control studies of epidemiological data [82–84, 87–92, 103–108, 114, 115], which led IC to recommend new vaccine tools (e.g. mOPVs, bOPV, IPV) as a way to get around poor programmatic performance. IC and IDM also both focused attention on applying statistical models to characterize population immunity (as they, respectively, defined it for different studies, see note at the bottom of Table 4) and on identifying national and subnational areas that previously performed poorly, for which they recommended temporary shifts or optimization of resources to deal with the failure to vaccinate in some populations [87, 89, 92, 93, 100–102, 109, 111, 131, 140–142]. The differences between the recommendations of the modeling groups with respect to the delays in achieving polio eradication as due to failure to vaccinate vs. due to vaccine failure led to substantially different foci and investments. KRI suggests that chasing better (and often more expensive) tools (e.g. mOPV, IPV) has not helped accelerate global polio eradication, that achieving and maintaining eradication depends on continuing to get enough OPV preventively into susceptible children to stop and prevent the transmission of cVDPVs and/or WPVs (followed by careful and aggressive management of the risks of globally coordinated OPV cessation), and that as of early 2020, the GPEI appears off track [202, 203].

### 5.7. Undetected circulation

Building on modeling performed prior to 2000 that supported the certification of the Americas as wild poliovirus free [197–199], multiple studies published in 2000–2019 explored the potential of undetected circulation and confidence about no circulation [27, 46, 70, 71, 75, 76, 132, 136, 161, 167–169]. Generally, the modeling studies to date agreed with respect to their recommendations about undetected circulation and high confidence about no circulation after 3 years with no detected evidence of LPV transmission while conducting high-quality surveillance. Although not captured in the review, modeling of one of the last known reservoirs of WPV3 transmission (i.e. Borno and Yobe, Nigeria) published in 2020 [233, 234] also supported the 2019 decision by the Global Certification Commission to certify the global eradication of indigenous WPV3 [235].

### 5.8. Environmental surveillance

As the GPEI expanded its use of environmental surveillance, the modeling groups published increasing numbers of studies that included consideration of the information that environmental surveillance provides [43, 46, 67, 71, 75, 76, 117, 136, 145, 147, 162, 166].

### 5.9. Other risks

To date, only KRI considered the risks of iVDPVs [4, 13, 50, 61, 81] and (un)intentional re-introduction risks (e.g. breaches in containment) in its global modeling (see Table 4 for references), although IC recently reviewed the WHO database of known iVDPVs [125].

## 6. Different types of studies and their limitations

This review highlights the different types of polio transmission models developed and applied. One independent study included extensive discussion about some of the differences and limitations of models published by the three modeling groups, in particular noting the complexity of the KRI model [165]. All models depend on the scope (i.e. boundaries of the system), assumptions about structure of the system and the causal relationships that determine the equations used and the selection of model inputs, and are limited by their assumptions [203]. This section highlights some of the key differences in and limitations of the different modeling approaches.

### 6.1. Dynamic, prospective, and integrated (with economics) models vs. statistical models on retrospective data or from controlled studies

KRI represents the only group that published integrated dynamic disease transmission and economic models that prospectively explore(d) the risks, costs, and benefits of strategies and policies to support the GPEI. By design, prospective models represent inherently uncertain projections into the future, and the results and insights from these models are only as good as the assumptions and the underlying available evidence. The KRI dynamic poliovirus transmission models [10, 33, 202] rely on using the available evidence and subject matter expert opinion to characterize the dynamics of poliovirus transmission as a function of differential equations, with consideration of some of the variability that exists among countries based on stratification by WBILs and relevant inputs related to transmission, seasonality, and actual vaccine use. KRI uses a model with high complexity and checks its models retrospectively to ensure that they provide estimates consistent with historical data of cases caused by WPVs and VDPVs, die out, and children with non-polio acute flaccid paralysis (NP-AFP) with a history of zero doses of vaccine, and then applies them prospectively to address policy and strategy questions [10, 33, 202]. The KRI poliovirus transmission and OPV evolution model include assumptions about a multi-stage infection process with infection stages of variable infectiousness that impacts the kinetics of infections and die-out and depends on choices about the number of stages used to model OPV evolution. These choices influence the flows of people and timing of transitions between reversion stages, while actual OPV evolution and the emergence of cVDPVs depend on random events and micro-level population dynamics [33, 202]

IDM developed and applied multiple IB models that also include considerable complexity. The first published IDM IB dynamic transmission model captures within-host susceptibility by exposure to and dose history for LPVs and/or IPV, models shedding durations and concentrations based on the host immunity histories, and assumes fecal-oral transmission among people who share a household as well as through close social contacts outside the household [129, 132, 133]. IDM applied an IB model to reproduce WPV viral shedding in different settings based on historical data [134] and added secondary spread of OPV, reinfection, and waning in some of its IB models [134, 135]. IDM does not model OPV evolution (i.e. the transition from Sabin OPV to cVDPVs) endogenously in its IB modeling [134], although Table 2 notes that IDM included statistical consideration of OPV reversion to cVDPV in one study [133]. IDM recently performed a cost study [146], but has not to date published any studies that integrated dynamic poliovirus transmission modeling with economics. IDM did not report substantial or prohibitive computational expense associated with following many individuals in IB models given the populations that it modeled to date, although IDM reported using a sampling strategy or reduced scope model to avoid computational burden in some of its IB modeling papers [132, 133].

IC did not consider economics in any of its modeling. As shown in Table 1, IC developed a few SC models and applied them prospectively to address specific questions. However, most of the publications by IC present statistical analyses of existing, retrospective data with a focus on answering specific questions driven by the data. Extrapolation of the results and inferences from statistical models requires assuming that the data collected in the past provide a good representation of the future and directly relate to the question asked. With eradication efforts driving cases to zero, epidemiological models lose their ability to make inferences based on comparing observed retrospective cases for different interventions, because as the polio cases disappear the data become sparse and controlling the data for confounders and other biases becomes difficult. The case–control epidemiological methods used by IC remain highly sensitive to the selection of cases and controls, and any limitations associated with the data used to perform the analyses.

In addition, in the context of complex dynamic systems, statistical models can provide relatively poor insight about prospective risks. For example, in the early 2000s, when countries only used tOPV (i.e. no mOPV or bOPV), KRI characterized the risks of cVDPVs using a statistical model [13]. However, a subsequent review of available data demonstrated the inadequacy of this approach following the introduction of mOPV and bOPV, which created substantial immunity gaps for serotype 2, and increased the risks of cVDPV2s [32], which led KRI to add OPV evolution endogenously into its dynamic transmission model [33, 202].

### 6.2. Different assumptions for modeling populations and mixing

As shown in Tables 2 and 3, the models reviewed differed with respect to the populations modeled and the mixing assumptions used. DEB and SC models typically assume homogeneous mixing of individuals in a population, although they may account for preferential mixing by age, subpopulation, or other factors, and include births, deaths, aging, and immigration. Part of the complexity of KRI transmission models comes from the use of population-specific demographic and immunization history data for inputs and the inclusion of preferential mixing by age and/or subpopulation. The inclusion of undervaccinated subpopulations in DEB models probably only partially captures some of the population heterogeneity in under-vaccinated communities, but does so better than ignoring this heterogeneity for some analytical questions.

IB models seek to capture the full richness of the complexity of transmission, but they do so with considerable computational costs. IB models track each individual in a population, which can offer advantages that include simulating die-out directly, but require many assumptions about the spatial distribution and contact patterns for each individual in the model [24, 41].

One limitation of transmission models broadly arises from assumptions about mixing at the model boundaries. Most models characterize transmission within a closed population, but they can allow for importations and exportations of viruses as appropriate [33, 202].

### 6.3. Different assumptions for die out

DEB models use simplified population structures and fractional rate-based processes that allow for fractional individuals, which requires the use of a transmission threshold to simulate die-out [10, 33, 202]. In real populations, the die out of transmission involves some element of chance. For analyses that focus on low levels of transmission and die out (e.g. analyses about the confidence of no undetected circulation), the modeling groups typically apply SC models to simulate the stochasticity of die out, although they have used both types of stochastic simulation approaches.

## 7. Conclusion

Recognizing that all models represent simplifications of reality, we suggest that the polio modeling performed during the past 20 years offered insights on many different aspects of the polio endgame that supported GPEI-partner deliberations and decisions. Although the models developed by independent groups took different approaches, they generally offered similar insights and recommendations. Notably, we found relatively few conflicts between the recommendations made by the modeling groups in the published literature, although the differences in recommendations about some vaccination strategies were substantial (e.g. initial recommendations about tOPV pSIAs in the run-up to OPV2 cessation, IPV use in oSIAs). The review suggests that some of the differences in recommendations may reflect different approaches and use of data. Both KRI and IDM developed comprehensive dynamic transmission model platforms, which they designed, adopted, and applied for use in addressing different questions. The broad and deep nature of polio dynamic transmission models led to the inclusion of significant complexity, which matches the human experience with polio: it is complicated. Notably, the large and multi-component KRI and IDM models required significant time to develop and require a time investment to fully understand. In general, model platforms can offer the advantages of internal consistency and consistency with all of the available evidence, to the extent that they are well calibrated and consider all of the evidence.

## 8. Expert opinion

The polio endgame appears far from over [202], and the potential for modeling to contribute to future polio risk management activities appears promising. The GPEI, now 20 years late in delivering on polio eradication, faces an uncertain path and future. During the next few years, the success or failure of the 2016 globally coordinated OPV2 cessation will become clear, and global health leaders will evaluate their commitments to OPV cessation as a polio endgame strategy [80]. Modeling published in early 2020 suggests that the GPEI remains off track with respect to achieving WPV1 eradication and successful OPV2 cessation [202], although WPV1 eradication remains possible with sufficiently high-intensity OPV vaccination [236, 237]. Further modeling can help to quantify the probability of needing to restart OPV2, which a 2020 statistical analysis [238] and modeling study suggest appears likely [239].

Modeling studies will need to reevaluate the health and economic impacts of the GPEI, if it succeeds, and evaluate the costs and benefits of future poliovirus vaccine options. In the case of a successful OPV cessation, all countries will realize the benefits of a world free of WPVs and cVDPVs, and determine their interest in purchasing the long-term insurance offered by IPV use. We expect that high- and upper middle-income countries will continue to use IPV and increasingly use IPV-containing combination vaccines, albeit at relatively high costs [240]. We also anticipate a continued trend toward the use of IPV-only schedules by these countries, although as long as LPVs continue to circulate, countries at risk of LPV importation will likely continue with sequential IPV/OPV schedules.

For relatively lower-income countries, polio vaccine choices appear more complicated. As the risks of continued transmission of LPV2s continue to pose threats to successful OPV2 cessation, questions will arise about OPV cessation as a strategy and about the need for different vaccine options for the polio endgame. Perhaps the most interesting role played by dynamic transmission and economic modeling arises from the opportunities they offer to explore potential future options [61–64]. After many years of research and development, a new and more difficult-to-revert OPV2 strain, which researchers expect will provide the protection of Sabin OPV but lower risks of VAPP and VDPVs, may become a real option [241]. The existence of such new OPV strains will raise important modeling questions about whether countries that currently use OPV will want to shift their polio immunization strategy to use trivalent formulations of such OPV strains, or continue to use IPV. The choices will depend substantially on the costs of the different vaccine options (including national costs of delivery), the risks posed by LPVs, and the effectiveness of the vaccines with respect to providing protection from paralysis and/or transmission. Thus, with respect to different polio vaccine options, the world will look very different if the GPEI succeeds in its efforts to eradicate all WPVs and coordinates global cessation of all OPV than a world with ongoing control using OPV due to continued WPV1 transmission.

Questions also remain about the formulations of IPV vaccine that may become available in the future, which will affect future poliovirus vaccine policy modeling and the path of the polio endgame [240]. Currently, most OPV-using countries deliver stand-alone formulations of IPV, although in some cases they use off-label fractional intradermal delivery, which can save substantially on antigen costs, but cost more with respect to administration [230]. Research underway may also provide an IPV vaccine patch option [242–245], which would potentially achieve both dose-sparing of IPV and ease of delivery that could increase coverage, albeit at a potentially higher and uncertain cost. Future modeling could support current efforts to explore greater use of combination vaccines that contain IPV (e.g. adding IPV to current pentavalent vaccines), which offer an opportunity to potentially save some vaccine administration costs by sharing across antigens. However, the actual costs for the IPV component itself may increase due to the added complexity of the vaccine. Moreover, national costs for IPV vaccination will also increase with the use of combination vaccines because the other vaccines in such combination products (i.e. DPT, Heb, and Hib), which require more doses and differ with respect to their ideal schedules (e.g. IPV shows better take rates when given to children after maternal antibodies wane, which occurs after scheduled DPT doses in most current OPV-using countries). To avoid giving extra doses of IPV, countries could adopt schedules that include both pentavalent and hexavalent vaccines, but this adds complexity for both supply chains and administration, which also implies additional costs. Furthermore, combination IPV products use full (i.e. not fractional) IPV doses, which implies no dose sparing. In addition to national preferences, we note that the mix of future poliovirus vaccine options available to developing countries will also depend on the extent to which donors who support immunization for lower-income countries prefer different vaccine options. Modeling may help to support future investment decisions, particularly since all of this complexity and uncertainty imply the potential for insufficient supplies of the desired vaccines given the time delays associated with producing vaccines.

Modeling the polio endgame and particularly the potential for OPV restart can also motivate the exploration of investments in developing new, non-vaccine risk management strategies for the polio endgame. For example, the results of modeling the reveal under-vaccinated subpopulations can lead to the development of better evaluation methods to monitor vaccine delivery (e.g. GPS tracking [246]), surveillance (e.g. GIS settlement mapping [247]), and/or other interventions. As with vaccines, the extent to which countries and donors are willing to invest in future risk management tools and strategies will determine their development pathway and ultimate use. The willingness to support modeling studies will also determine the demand and resources available for future modeling.

Finally, the COVID-19 virus pandemic will motivate further polio modeling to support the recovery of GPEI functions and options for managing financial and vaccine resources. Modeling also may help the GPEI partners evaluate the combined impacts of the physical distancing efforts made by individuals in some countries, which change mixing and the dynamics of poliovirus transmission, and reduced health system utilization, which decreases the distribution of polio vaccines in RI and/or SIAs and may decrease surveillance.

## Article highlights

- Systematic review of the literature demonstrates substantial contributions from polio models published in 2000–2019.
- The complexities of polioviruses and the two poliovirus vaccines make policies and modeling challenging.
- Different types of models, scopes, and definitions can lead to conflicting recommendations.
- Long-term poliovirus vaccine use remains uncertain as the polio endgame extends further in time.
- Future polio modeling offers opportunities to evaluate the risks, costs, and benefits of risk management options, including vaccines.
